# Prevalence and predictors of kaposi sarcoma herpes virus seropositivity: a cross-sectional analysis of HIV-infected adults initiating ART in Johannesburg, South Africa

**DOI:** 10.1186/1750-9378-6-22

**Published:** 2011-11-17

**Authors:** Mhairi Maskew, A Patrick MacPhail, Denise Whitby, Matthias Egger, Carole L Wallis, Matthew P Fox

**Affiliations:** 1 Clinical HIV Research Unit, Department of Medicine, Faculty of Health Sciences, University of the Witwatersrand (York Avenue), Johannesburg (2193), South Africa; 2 Health Economics and Epidemiology Research Office, Department of Medicine, Faculty of Health Sciences, University of the Witwatersrand (York Avenue), Johannesburg (2193), South Africa; 3 Right to Care (Perth Road), Johannesburg (2192), South Africa; 4 Viral Oncology Section, AIDS and Cancer Virus Program, SAIC-Frederick, NCI-Frederick (Rosemont Avenue), Frederick MD (21702-1201), USA; 5 Division of International and Environmental Health, Institute of Social and Preventive Medicine (ISPM), University of Bern (Finkenhubelweg), Bern (3012), Switzerland; 6 Department of Molecular Medicine and Hematology, Faculty of Health Sciences, University of the Witwatersrand (York Avenue), Johannesburg (2193), South Africa; 7 Center for Global Health and Development, Boston University (Massachusetts Avenue), Boston (02118), USA; 8 Department of Epidemiology, Boston University School of Public Health (Massachusetts Avenue), Boston (02118), USA

**Keywords:** Kaposi sarcoma, Kaposi sarcoma herpes virus, resource-poor setting, antiretroviral therapy

## Abstract

**Background:**

Kaposi sarcoma (KS) is the most common AIDS-defining tumour in HIV-infected individuals in Africa. Kaposi sarcoma herpes virus (KSHV) infection precedes development of KS. KSHV co-infection may be associated with worse outcomes in HIV disease and elevated KSHV viral load may be an early marker for advanced HIV disease among untreated patients. We examined the prevalence of KSHV among adults initiating antiretroviral therapy (ART) and compared immunological, demographic and clinical factors between patients seropositive and seronegative for KSHV.

**Results:**

We analyzed cross-sectional data collected from 404 HIV-infected treatment-naïve adults initiating ART at the Themba Lethu Clinic, Johannesburg, South Africa between November 2008 and March 2009. Subjects were screened at ART initiation for antibodies to KSHV lytic K8.1 and latent Orf73 antigens. Seropositivity to KSHV was defined as positive to either lytic KSHV K8.1 or latent KSHV Orf73 antibodies. KSHV viremia was determined by quantitative PCR and CD3, 4 and 8 lymphocyte counts were determined with flow cytometry. Of the 404 participants, 193 (48%) tested positive for KSHV at ART initiation; with 76 (39%) reactive to lytic K8.1, 35 (18%) to latent Orf73 and 82 (42%) to both. One individual presented with clinical KS at ART initiation. The KSHV infected group was similar to those without KSHV in terms of age, race, gender, ethnicity, smoking and alcohol use. KSHV infected individuals presented with slightly higher median CD3 (817 vs. 726 cells/mm^3^) and CD4 (90 vs. 80 cells/mm^3^) counts than KSHV negative subjects. We found no associations between KSHV seropositivity and body mass index, tuberculosis status, WHO stage, HIV RNA levels, full blood count or liver function tests at initiation. Those with detectable KSHV viremia (n = 19), however, appeared to present with signs of more advanced HIV disease including anemia and WHO stage 3 or 4 defining conditions compared to those in whom the virus was undetectable.

**Conclusions:**

We demonstrate a high prevalence of KSHV among HIV-infected adults initiating ART in a large urban public-sector HIV clinic. KSHV viremia but not KSHV seropositivity may be associated with markers of advanced HIV disease.

## Background

Since there has been greater access to antiretroviral therapy (ART) [1-3], increased longevity among those infected with HIV has made morbidity and mortality from cancers associated with HIV increasingly more common [4]. Viral associated cancers including cervical cancer, non-Hodgkin's lymphoma and Kaposi sarcoma (KS) are prominent among HIV-infected individuals [4,5]. Kaposi sarcoma is the most common tumour in HIV-infected individuals in Africa and is preceded by infection with Kaposi sarcoma herpes virus (KSHV) [6]. Kaposi sarcoma was relatively common in South Africa (up to 5 per 1000 population at risk) prior to the AIDS epidemic [7] but the incidence increased dramatically as the epidemic escalated [4,5,8]. The incidence of KS has decreased in the US and Europe with the introduction of HAART [9-11] but the impact of HAART in Africa where the underlying prevalence of KSHV is higher is yet to be determined.

While clinical Kaposi sarcoma is known to be a marker of advanced HIV disease and is one of the WHO stage 4 and AIDS-defining illnesses [12], it is unclear if co-infection with oncogenic viruses such as KSHV places untreated HIV-infected patients at similar risk even without clinically apparent illness. Several clinical and laboratory markers have been associated with advanced disease stage among untreated HIV-infected individuals [13-21], including the T-lymphocyte subpopulations, CD4+ and CD8+ [14,18,22-26] which play an important role in the response to viral infections. While KSHV-specific CD8+ T cell epitope responses have been shown to increase after initiation of HAART [27,28], it has yet to be determined if T-lymphocyte subpopulations are also a marker of advanced disease stage in ART naive patients infected with KSHV (as seen among the HIV-infected population) and if this has implications for treatment initiation guidelines.

We enrolled subjects in a cohort study to determine the impact of KSHV on response to HAART as well as the effects of HAART on KSHV control. This cross-sectional analysis forms part of this larger study and aimed to measure the prevalence of KSHV infection among these HIV-infected adults initiating ART in a large treatment programme in Johannesburg, South Africa and to compare T-lymphocyte subpopulations and other demographic, clinical and laboratory factors between patients seropositive and seronegative for KSHV at enrolment.

## Methods

### Study design

This cross-sectional study utilized data from patients enrolled in care at the Themba Lethu Clinic in Johannesburg, South Africa. Currently, Themba Lethu has over 23,000 HIV infected adults enrolled in its comprehensive HIV care, management and treatment program. Since inception, over 16,000 of these patients have been initiated on ART at this clinic. Care at the clinic is provided according to the guidelines from the South African National Department of Health [29]. Patient data at Themba Lethu is captured and stored in an electronic patient record, TherapyEdge-HIV™. At enrolment into care, data on demographics, physical examination and clinical diagnoses are recorded. At initiation of ART, laboratory test results, including CD4 lymphocyte counts, full blood counts and liver function tests, are also recorded.

### Eligibility Criteria

Between November 2008 and March 2009, all HIV-positive treatment naïve patients > 18 years of age who met the National guidelines criteria for initiation of ART (CD4 count < 200 cells/mm^3 ^or WHO stage 4 defining illness) and who were attending group counselling sessions at Themba Lethu were invited to participate in the study. Patients who did not meet these criteria or had a history of prior ART use were excluded from the study.

### Study variables

Venous blood samples were drawn from all study participants prior to initiation of ART to determine KSHV serostatus. Seropositivity to KSHV was defined as a positive reaction to either lytic KSHV K8.1 or latent KSHV Orf73 antibodies detected using enzyme-linked immunosorbent assays (ELISA). Detection of antibodies to a single antigen has been shown to potentially underestimate the prevalence of KSHV, therefore, the ELISA to detect antibodies to latency associated nuclear antigen was performed in addition to the lytic K8.1 ELISA to provide a more accurate assessment of KSHV antibody status [30]. KSHV viremia was determined using 150 ng of DNA extracted from buffy coat using the QIAamp DNA Blood Midi kit, according to the manufacturer's instructions. Quantitative TaqMan PCR as per previously published methodology [31] was performed on the ABI Prism 7900 sequence detection system (Applied Biosystems, Forster City, CA). Subject and control samples were run in triplicate. The KSHV viral load assay has a linear dynamic range of 8 logs and is calibrated to detect a single copy of viral DNA in 150 ng genomic DNA. CD3, CD4 and CD8 lymphocyte counts (components of the total lymphocyte count) were performed using flow cytometry and standard methodology.

Additional data was extracted from the electronic patient record. Demographic variables of interest included gender, age at study enrolment, race, as well as ethnicity (using mother and father tongue as a proxy). Clinical data on initiating ART regimen, WHO clinical stage, body mass index (BMI), tuberculosis status and HIV RNA level at enrolment were also extracted as well as laboratory results for full blood counts and liver function tests.

### Statistical analysis

The prevalence of KSHV among the study group was estimated and is presented with corresponding 95% confidence bounds. Demographic, clinical and laboratory characteristics of the participants at study enrolment were stratified by KSHV status and summarized as simple proportions or medians with interquartile ranges (IQR). Logarithmic transformations of optical densities for lytic K8.1 and latent Orf73 were performed and are presented as geometric means. Crude prevalence ratios for participant presenting features were estimated using log-binomial regression models stratified by KSHV serostatus. This analysis was also further stratified by reaction to lytic K8.1 or latent Orf73 antigen. Models were adjusted for age, sex and presenting CD4 count where appropriate.

Approval to conduct this study and use of data from the Themba Lethu site was granted by the Human Research Ethics Committee of the University of the Witwatersrand.

## Results

All eligible subjects initiating ART at Themba Lethu Clinic between November 2008 and March 2009 were invited to participate and we enrolled 404 of these. Response rates were high: it is estimated that fewer than 5% of those invited to participate refused. However, in order to determine if the sample was representative of the untreated population accessing HIV care at that time, we compared this sample to the general population presenting for treatment initiation at Themba Lethu during the recruitment period that were either not invited or refused to participate in the study. The presenting features of the study group were very similar to the Themba Lethu Clinic general population at ART initiation (Table 1) in terms of age, CD4 cell count, HIV viral load, proportion with WHO stage 3 or 4 defining illness, hemoglobin level and BMI. The Themba Lethu Clinic general population had a slightly lower proportion of females (61% vs. 65%) and slightly lower proportion presenting with tuberculosis (11% vs. 14%) compared to the study group.

**Table 1:** Presenting features of 404 ART naïve adults in care at Themba Lethu in Johannesburg, South Africa stratified by KSHV^&^status

**Characteristics**^**#**^		* General Clinic Population (n = 679)	**KSHV+**^**&**^**(n = 193)**	**KSHV-**^**&**^**(n = 211)**
Female		416 (61%)	127 (66%)	135 (64%)

Age (yrs.)	Median (IQR)	37 (31-43)	37 (32-47)	38 (32-45)

WHO Stage	I/II	408 (64%)	116 (64%)	114 (61%)
(n, %)	III/IV	231 (36%)	64 (36%)	74 (39%)

BMI	< 18.5	118 (23%)	38 (21%)	57 (29%)
(n, %)	18.5-24.9	286 (56%)	98 (56%)	94 (48%)
	25-30	72 (14%)	23 (13%)	31 (16%)
	> 30	37 (7%)	15 (8%)	14 (7%)

Hemoglobin (n, %)	≤ 8.0 g/dL	41 (7%)	14 (7%)	13 (6%)

CD4 count (cells/mm^3^)	Median (IQR)	100 (39-173)	101 (46-168)	89 (34-164)

CD4 cell count category	0-50	167 (31%)	47 (28%)	63 (34%)
(n, %)	51-100	106 (19%)	39 (23%)	39 (21%)
	101-200	182 (33%)	65 (38%)	56 (30%)
	200-350	89 (16%)	20 (12%)	30 (16%)

Tuberculosis (n, %)	Yes	75 (11%)	26 (14%)	30 (15%)

HIV RNA	Median (IQR)	20000 (8300-39000)	15500 (6400-33500)	19500 (7300-39000)

^# ^Characteristics at eligibility for initiation of antiretroviral therapy

* Themba Lethu clinic general population refers to treatment naïve adults eligible for initiation of antiretroviral therapy during the study period who were not recruited into the study

^&^KSHV = Kaposi sarcoma herpes virus

The median age of the study group was 38 years (IQR 32-45 years) and 262 (65%) were women. The median CD4 count at ART initiation was 87 cells/μL (IQR 40-149 cells/μL) and nearly 40% presented with a WHO stage III/IV defining condition. The majority of participants were started on standard public-sector first-line ART regimens: 86% on stavudine, lamivudine and efavirenz and 7% on stavudine, lamivudine and nevirapine. The remaining 7% who presented with a contra-indication to one of the standard first-line regimens were initiated on zidovudine, lopinavir/ritonavir or tenofovir-based regimens.

### Prevalence of KSHV

Among the study participants, 193/404 tested positive to lytic KSHV K8.1 and/or latent KSHV Orf73 antibodies at ART initiation; the prevalence of KSHV in this urban population was estimated at 48% (95%CI: 43-53%). Of those positive for KSHV, 76 (39%; 95% CI 33-46%) were reactive to lytic K8.1 alone, 35 (18%; 95% CI13-24%) to latent Orf73 and 82 (42%; 95%CI 36-50%) to both. Only one individual presented with clinical KS at ART initiation. This individual was positive to both lytic and latent KSHV antigen but did not have a detectable KSHV viral load.

### KSHV viral load

Of the 193 individuals serologically positive to KSHV, 167 (87%) had samples available for KSHV viral load testing. KSHV DNA was detected in 19 (11%) of the buffy coat samples of those serologically positive to KSHV with a median of 10.6 (IQR 6.7-37.5) copies/uL and values ranging from 1.91 - 109.8 copies/uL. A higher proportion of those positive to both lytic and latent KSHV antibodies had detectable KSHV viral loads (20%) compared to those positive to only lytic (3%) or only latent (8%). The estimates suggested that those with a detectable KSHV viral load were more likely to be male (PR = 1.43; 95%CI 0.61-3.35), have a WHO stage III/IV illnesses (PR = 1.51; 95%CI 0.65-3.50) and hemoglobin ≤ 8 g/dL (PR = 1.42; 95%CI 0.37-5.43) compared to those without a detectable KSHV viral load, although these results lacked precision (as indicated by the wide confidence intervals).

### Associations with overall KSHV seropositivity

**Figure 1:**
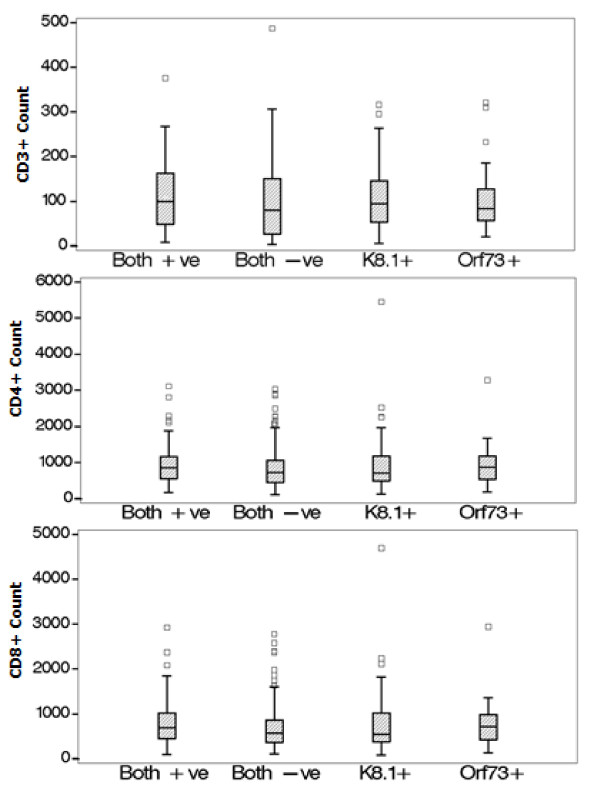
**Box Plots of CD3+ (top), CD4+ (middle) and CD8+ (bottom) lymphocyte counts by KSHV serostatus**.

The KSHV infected group was similar to those without KSHV group in terms of age, race, gender and ethnicity (mother and father tongue). KSHV positive individuals presented with slightly higher median CD3 (817 vs. 726 cells/mm^3^) and CD4 (90 vs. 80 cells/mm^3^) than KSHV negative subjects (Figure 1). In log-binomial regression models, adjusted prevalence ratios of KSHV seropositivity was increased for those with CD3 and CD8 counts ≥ 500 cells compared to those < 500 cells and also CD4 counts between 51 and 200 cells compared to CD4 counts ≤ 50, (Table 2) although some of these estimates lacked precision (i.e. our confidence intervals are wide). Those with a BMI > 18.5 kg/m^2 ^were also more likely to be infected with KSHV while those with Zulu as mother tongue were somewhat less likely to be KSHV positive. We found no association between KSHV seropositivity and several other factors including gender, age, WHO stage and tuberculosis status. Estimates were adjusted for gender, age and baseline CD4 count, where appropriate.

**Table 2:** Associations with lytic/latent KSHV^# ^seropositivity

	**Overall KSHV**^# ^**positive^€^**		Positive to Lytic K8.1 only^¥^		Positive to Latent Orf 73 only^β^	
	
	Total Positive (n, %)	Adjusted PR^# ^(95%CI)	Total Positive (n, %)	Adjusted PR^#^ (95%CI)	Total Positive (n, %)	Adjusted PR^# ^(95%CI)
Gender						
Female	127 (66%)	1	104 (66%)	1	73 (62%)	1
Male	66 (34%)	0.96 (0.77-1.19)	54 (34%)	0.98 (0.75-1.26)	44 (38%)	1.16 (0.84-1.60)

Age category						
< 40 yrs.	118 (61%)	1	94 (59%)	1	69 (60%)	1
≥ 40 yrs.	75 (39%)	1.06 (0.86-1.31)	64 (41%)	1.00 (0.78-1.29)	48 (41%)	0.99 (0.73-1.35)

Language						
Zulu	43 (32%)	1	35 (33%)	1	29 (35%)	1
Other	90 (68%)	1.21 (0.93-1.58)	71 (67%)	1.17 (0.84-1.62)	54 (65%)	1.09 (0.75-1.59)

Has tuberculosis						
No	160 (86%)	1	131 (87%)	1	97 (87%)	1
Yes	25 (14%)	1.06 (0.78-1.42)	20 (13%)	1.08 (0.74-1.57)	15 (13%)	1.06 (0.67-1.67)

BMI category						
< 18.5	36 (21%)	1	28 (20%)	1	20 (19%)	1
≥ 18.5	137 79%)	1.28 (0.97-1.70)	114 (80%)	1.33 (0.94-1.90)	85 (81%)	1.46 (0.94-2.26)

Hemoglobin						
≥ 8.5 g/dL	166 (89%)	1	136 (89%)	1	96 (85%)	1
< 8.5 g/dL	21 (11%)	1.15 (0.84-1.56)	17 (11%)	1.17 (0.80-1.72)	17 (15%)	1.64 (1.11-2.43)

CD4 count						
0-50 cells	47 (28%)	1	39 (28%)	1	24 (23%)	1
51-100 cells	39 (23%)	1.18 (0.87-1.62)	29 (21%)	1.03 (0.68-1.55)	28 (27%)	1.61 (0.97-2.66)
101-200 cells	65 (38%)	1.28 (0.97-1.68)	53 (38%)	1.16 (0.75-1.81)	37 (36%)	1.29 (0.72-2.30)
201-350 cells	19 (11%)	0.98 (0.65-1.47)	17 (12%)	0.96 (0.52-1.76)	15 (14%)	1.27 (0.59-2.90)

CD3 count						
< 500 cells	41 (21%)	1	34 (22%)	1	22 (19%)	1
≥ 500 cells	152 (79%)	1.35 (1.04-1.76)	124 (78%)	1.31 (0.93-1.84)	95 (81%)	1.62 (1.04-2.54)

CD8 count						
< 500 cells	64 (33%)	1	54 (34%)	1	35 (30%)	1
≥ 500 cells	129 (67%)	1.20 (0.96-1.50)	104 (66%)	1.09 (0.81-1.47)	82 (70%)	1.41 (0.96-2.09)

# KSHV = Kaposi sarcoma herpes virus, PR = prevalence ratio estimated from log-binomial regression model. All models adjusted for sex, age and baseline CD4 count

**^€ ^**KSHV negative individuals used as the comparison group

¥ Those who did not react to lytic K8.1 including the KSHV negative individuals used as the comparison group

**^β ^**Those who did not react to latent Orf73 including the KSHV negative individuals used as the comparison group

### Associations with lytic or latent antibody presence

The geometric mean of the optical density was 0.83 (95%CI 0.80-0.87) for lytic K8.1 and 0.34 (95%CI 0.32-0.37) for latent Orf73. Presence of antibodies to latent Orf73 was associated with higher median CD3 (863 vs. 720 cells/mm^3^; p = 0.019) and CD8 (706 vs. 569 cells/mm^3^; p = 0.026) cell counts. Higher BMI (≥ 18.5 kg/m^2^) was associated with reactivity to both lytic K8.1 [PR = 1.33 (0.94-1.90)] and latent Orf73 [PR = 1.46 (0.94-2.26)] in models adjusted for age, gender and CD4 cell count (Table 2), while higher CD3, CD4, CD8 counts as well as a low hemoglobin level were associated more strongly with reactivity to latent Orf73 than lytic K8.1.

## Discussion

The prevalence of KSHV is reported to be high in Africa [32-34]. Those co-infected with KSHV and HIV are at high risk for Kaposi sarcoma, a condition associated with poor outcome in HIV-infected patients. This cross-sectional analysis estimated the prevalence of KSHV among adults initiating ART at a large urban clinic in Johannesburg and investigated factors associated with KSHV seropositivity and reactivity to either lytic or latent KSHV antigen. We demonstrated a high prevalence of KSHV in this population and found that few factors associated with advanced HIV disease stage and progression were also associated with KSHV seropositivity. In fact, reactivity to latent KSHV antigen was associated with better markers of immunity in terms of higher CD3, CD4 and CD8 cell counts.

The high prevalence of KSHV (48%) demonstrated in this setting is comparable with findings from similar settings among HIV-infected populations [34,35]. In contrast, KSHV prevalence appears to be much lower in the developed world among the HIV uninfected but higher among pregnant women [34] and men who have sex with men [36]. These findings have fuelled theories of a sexual route of transmission for KSHV [37]. Such conflicting results are probably the result of varying routes of transmission of this virus and much geographic variability in prevalence rates [38]. A South African study showed rates of KSHV infection to vary from 35% to 49% across different municipalities within one province [34]. Among populations with a high prevalence of KSHV seropositivity, the route of infection is likely to be saliva and is acquired during childhood and early adult life [39,40]. Sexual and non-sexual transmission seems to occur in KSHV naïve adult populations such as men who have sex with men and sex-workers [36,37,41,42].

Advanced age, male gender, nutrition, anemia, concurrent tuberculosis and other opportunistic infections have been demonstrated to be associated with an advanced stage of HIV disease and mortality among untreated individuals [13,14,16,17,19-22,37]. Our finding of a positive association between anemia and KSHV seropositivity among the group reactive to latent Orf73 is in keeping with this. However, we found no evidence of an association with other poor prognostic features; in fact the KSHV positive group had higher BMI and CD3, 4 and 8 cell counts than their KSHV negative counterparts. Among the KSHV infected individuals, however, those with a detectable KSHV viral load presented with signs of more advanced HIV disease including anemia and WHO stage 3 or 4 defining conditions compared to those in whom the virus was undetectable. KSHV viremia has previously been associated with the likelihood of development of clinical Kaposi sarcoma and other signs of advanced HIV disease including thrombocytopenia and higher HIV viral loads [43]. KSHV DNA in plasma has also been shown to predict death among those with clinical Kaposi sarcoma [44]. Detectable KSHV viremia may indicate poor immune control of KSHV infection [43-45] which in turn may indicate more advanced HIV disease. Furthermore, KSHV viremia has been associated with increased risk of clinical Kaposi sarcoma among HIV infected subjects [45] and, therefore, HIV/KSHV co-infected subjects with detectable KSHV viral load may benefit from specific prophylactic strategies and increased monitoring for KSHV related diseases.

The optical density for lytic and latent KSHV was comparable to other work in similar settings with high HIV prevalence [34]. The optical density for lytic K8.1 KSHV antigen was higher than that for latent Orf73 and more individuals were reactive to the lytic antigen. This is in keeping with the theories that lytic antigen represents actively replicating virus, as would be seen in the case of advanced HIV disease, and poor control of the immune system in the untreated individual [28]. In addition to this, in our data, those positive to latent Orf73 antigen presented with markers of less immune suppression and less advanced disease. Those with higher BMI, higher CD3, 8 and 4 cell counts were more likely to be reactive to latent Orf73 than those who did not react to latent Orf73 (including the overall KSHV negative group). CD4 and 8 cells play important roles in cell-mediated immunity and control of viremia in the HIV-infected individual [46,47] and low absolute numbers of CD4 and 8 cells have been associated with an increased risk of disease progression and mortality among HIV-infected persons [18,22-25].

Co-infection with other viruses such as hepatitis, Epstein-Barr and human papilloma has been shown to increase the risk of HIV disease progression and mortality in immune suppressed HIV infected individuals [48-51]. There is plausible biological evidence that suggests KSHV could impact on disease progression through stimulation of HIV *tat *proteins and activation of HIV replication [52]. Despite this, the KSHV seropositive group in this study was associated with less immune suppression and better BMI, particularly among those seropositive to latent Orf73, an antigen expressed less often during active replication of the KSHV virus. One study among a cohort of long term non-progressors also found no effect of KSHV infection on persistence of long term non-progressor status [53].

Our findings must be considered in light of possible limitations of the study. Firstly, due to the cross sectional nature of the analysis, we cannot make inferences about causal relationships between KSHV and the factors under investigation. As KSHV transmission has been shown to occur even early in life [39,40], presumably by saliva, temporal relationships between KSHV infection and the factors considered are difficult to establish. However, information about possible associations between KSHV seropositivity and other known risk factors for HIV disease progression are useful in generating hypotheses about possible interactions between these viruses. Secondly, it is possible that the association we demonstrate between less suppression of the immune system and KSHV seropositivity is due, at least in part, to survival bias. If KSHV infection is, in fact, associated with more advanced immune suppression and subsequent faster HIV disease progression, one could expect those with KSHV to be at greater risk of mortality before being able to access HIV treatment and care. Thus, the KSHV population presenting for treatment may represent a particularly healthy group of KSHV-infected individuals who have survived to that point as suggested by higher BMI and T-lymphocyte subpopulations noted. Additionally, despite this high prevalence of KSHV in an immunologically suppressed population, only one individual presented with clinical Kaposi sarcoma. As national guidelines change and ART is initiated at higher CD4 counts, we may see changes in prevalence and presenting features of those co-infected with KSHV. Studies focused on HIV-infected individuals who are not yet eligible for ART and ideally among a population who have recently HIV seroconverted may shed light on this point. Thirdly, in light of the geographic and population differences in prevalence and routes of transmission of KSHV, it may not be possible to generalize these results to other populations, such as those in the developed world.

## Conclusions

Despite these limitations, this study offers some insight into, as yet, unanswered questions around the clinical effect of KSHV co-infection. We demonstrate a high prevalence of co-infection with KSHV among a group of treatment naïve, HIV-infected adults initiating ART at a large urban clinic in Johannesburg, South Africa. The study population was similar to the general population accessing care at Themba Lethu Clinic with respect to presenting features. The results may, therefore, be reasonably extrapolated to HIV positive individuals accessing care at urban public sector clinics in South Africa. Although there appeared to be some immunological differences in terms of CD3, CD4 and CD8 cell counts between the KSHV positive and negative groups, KSHV seropositivity was not associated with other known risk factors for disease progression such as age, gender and concurrent tuberculosis infection among the HIV-infected untreated population. If we assume that KSHV co-infection has little or no impact among the untreated population, it may follow that KSHV co-infection would have little effect on outcomes among treated individuals, unless KSHV has a large direct impact on the effectiveness of antiretrovirals. Future research efforts in this field should be focused on longitudinal studies to determine if KSHV seropositivity, particularly viremia, poses a risk for HIV treatment outcomes, development of KS immune reconstitution inflammatory syndrome and mortality in the presence of antiretroviral therapy.

## Competing interests

The authors declare that they have no competing interests.

## Authors' contributions

All authors contributed to the conception and design of the study. MM, PM, DW and CW contributed to acquisition of data. MM performed the statistical analysis. MM, MF, DW and CW interpreted the results and MM, MF, PM and ME drafted the manuscript. All authors revised the manuscript critically for intellectual content and have approved the submitted version.
